# The Resistance to Plague Infection among *Meriones persicus* from Endemic and Non-endemic Regions in Iran: The Role of Gut Microbiota

**Published:** 2018-01

**Authors:** Mehdi ASSMAR, Marjan KEYPOUR, Mehdi ROHANI, Ehsan MOSTAFAVI, Dariush DANESHVAR FARHUD

**Affiliations:** 1. Dept. of Parasitology, Pasteur Institute of Iran, Tehran, Iran; 2. Dept. of Epidemiology and Biostatistics, Research Center for Emerging and Reemerging Infectious Diseases, Pasteur Institute of Iran, Tehran, Iran; 3. National Reference Laboratory for Plague, Tularemia and Q Fever, Research Centre for Emerging and Reemerging Infectious Diseases, Pasteur Institute of Iran, Hamadan, Iran; 4. Dept. of Microbiology, Pasteur Institute of Iran, Tehran, Iran; 5. School of Public Health, Tehran University of Medical Sciences, Tehran, Iran; 6. Dept. of Basic Sciences/Ethics, Iranian Academy of Medical Sciences, Tehran, Iran

**Keywords:** Gut microbiota, Infectious disease, *Meriones persicus*, Plague, Iran

## Abstract

**Background:**

The present study was conducted approximately 40 years ago, but its results have not been released. At the time of this study, the importance of the gut microbiota was not fully understood.

**Methods:**

*Meriones persicus* rodents, as one of the major reservoirs of *Yersinia pestis* bacterium in Iran, were compared in a disease endemic area (Akanlu, Hamadan, western Iran) and a non-endemic zone (Telo, Tehran, Iran) from 1977 to 1981.

**Results:**

This study was able to transmit the resistance to *Y. pestis* to other rodents creatively by using and transferring gut microbiota.

**Conclusion:**

The study indicated for the first time that the gut microbiota could affect the sensitivity to plague in *Meriones* in Telo.

## Introduction

Forty years ago, the role of intestinal bacteria, nowadays known as the gut microbiota, in sensitivity of the rodents to plague was studied. In recent years, many studies have evaluated the role of gut microbiota on the pathogens transfer through vectors (1), their survival in carriers (2) and their character in human infection (3–5), which could increase our knowledge about this functional organ.

Generally, plague is a zoonotic disease and transmitted by fleas among wild rodents. Major epidemics of plague have occurred causing the death of many people. Plague is still an endemic disease in a number of countries in Asia, Africa, North and South America. Kurdistan Province in Iran is one of the endemic regions of plague. *Meriones persicus*, *M. libycus*, *M. tristrami*, and *M. vinogradovi* are the main plague-transmitting rodents in Iran. These rodents led to the epidemics occurred in 1964–1965 in the western regions of Iran mainly, Kurdistan and western Azerbaijan (6). Among these reservoir rodents, *M. persicus* and *M. libycus* are relatively resistant to plague, while *M. tristrami* and *M. vinogradovi* are sensitive to *Yersinia* infection (7). The relative stability of plague in resistant *Meriones* plays an important role during the episodes of plague bacillus maintaining (6).

Although *M. persicus* is the main reservoir of the disease in Kurdistan (Akanlu) and eastern Azerbaijan (Sarab) and has been also observed in Tehran, the plague infection has not been reported in non-endemic regions such as Tehran. This hypothesis was raised in 1953 that this could be due to the relative plague-resistance of *M. persicus* in plague endemic regions of Iran (8).

Therefore, the aim of this study was to find the factors making *M. persicus* in the Kurdistan region resistant to *Yersinia pestis* (8). This study conducted whether the resistance of *M. persicus* in Kurdistan is related to the intestinal bacteria flora of the wild rodents in this region, or to other factors such as genetic diversities and morphological characteristic.

## Materials and Methods

### Study Area

This cross-sectional study was performed on the *M. persicus* of two areas: Kurdistan, as an endemic region of plague, and Tehran, as a non-endemic region, from 1977 to 1981.

The sampling in Tehran was done in the Telo area located in the northeastern part of Tehran, capital of Iran. The sampling in Kurdistan was done in areas near Akanlu village that located in 365km distance from Tehran in the western part of the country. The rodents were trapped by wooden mousetrap for two months.

### Rodent identification

The morphological identification of the rodents was conducted by measuring the length of their head and body, the size of their ear, tail, and rear legs, and the color of their hair and incisors (9, 10). To compare the physiological characteristics of the rodents, their body temperature, heart rate, respiratory frequency, and feed rate were assessed for 24 h. To compare the chromosomal differences, 1 ml (per 100 gr of body weight of the *Meriones*) of 25% diluted solution of colchicine was injected intraperitoneally; the rodents were killed after one hour, and the femur was removed to study the chromosomes (11–13). The number of chromosomes and their connection position, i.e. metacentric, sub-metacentric, and acrocentric manners were then recorded.

### Blood biochemistry

The blood groups were evaluated by comparing the methods of non-insiders (hetero-agglutination) and insiders (isoagglutination) (14). The immunity acquired was evaluated by passive micro-haemagglutination assay (15–17). A total number of 75 *Meriones* serums were compared in both regions. The serum proteins of Meriones, including albumin, alpha-2-globulin, and beta and gamma globulin obtained from Akanlu and Telo regions were evaluated by the electrophoresis method (18–20); furthermore, the slides of the serum proteins and their curves were compared with the human serum proteins. The alpha-1-antitrypsin in *M. persicus* in the Akanlu region was compared with that of the Telo region via isoelectrofocusing on the polyacrylamide gel (21), using human serum samples as a control. The haptoglobin variety was also evaluated by starch gel electrophoresis method at the two regions (22). The transferrin variety was also studied, using high-voltage thin-layer agarose gel electrophoresis (23). Human serum was employed as a standard control. Moreover, ceruloplasmin heterogeneous and immunoglobulin A and G were evaluated by quantitative method, i.e. gel diffusion and tri-partigen plates (24–26). The base of these methods is antigen-antibody reactions. Ceruloplasmin and immunoglobulin were separately injected into adult rabbits to prepare anti-ceruloplasmins and anti-immunoglobulin. Then the serum samples of *Meriones* at the two regions were evaluated by anti-ceruloplasmins and anti-immunoglobulin. Isolation and purification of ceruloplasmin and immunoglobulin were performed and measured via ion exchange chromatography and biuret method, respectively.

The electrophoretic patterns of acid phosphatase (AP) (27), esterase D (ESD) (28), 6-phosphogluconate dehydrogenase (6-PGD) (29), phosphoglucomutase-1 (PGM-1) (30), and adenylate kinase (AK) (31, 32) enzymes in red blood cells in the two groups of *Meriones* were compared by thin-layer starch gel electrophoresis method and these enzymes in red blood cells were investigated for genetic polymorphisms.

### Parasitology

Ectoparasite and intestinal parasites were also evaluated in both areas. Ticks and fleas were removed from the body of the *Merione*s by forceps and observed by microscope (33, 34). The morphology of intestinal parasites was examined after opening a part of the intestine of *Meriones* and its staining with hemalum and Carmine-Alum (35, 36).

### The resistance of Meriones to plague infection (with and without gut microbiota exchange)

The sensitivity of *Meriones* to *Yersinia pestis* was investigated by *Y. pestis* PKR 775 strains obtained from Pasteur Institute (37). After preparing the microbial solution in different dilutions (the initial concentration was equal to 10^8^ CFU/ml), the *Meriones* of each region were divided into six groups, each including 10 *Meriones*. Then, 2 ml of each diluted microbial solution was injected subcutaneously; the *Meriones* was transferred to a laboratory and were isolated in plastic cages for 30 d. During this time, the dead *Meriones* were autopsied, and the blood smear was stained with Toluidine blue and studied by microscope. *Y. pestis* infection was also studied by culturing the spleen sample on blood agar plates.

The intestinal microbial flora of the *Meriones* was also investigated (38). For this purpose, the *Meriones* were killed. Afterwards, a part of their intestine was removed under sterile conditions, and their intestinal contents were cultured at 37 °C for 24 h on agar mediums. Each of the grown colonies was cultured on blood agar and MacConkey agar. The bacteria were identified by standard biochemical techniques and confirmed by serological tests, using specific microbial antibodies. Furthermore, suspensions equal to 3×10^8^ CFU/ml were prepared from the intestinal bacteria of the *Meriones* in the Akanlu region that did not exist in the *Meriones* in the Telo region; additionally, they were fed by syringe to the Telo group that had 12 *Meriones.* In addition, a group of 12 *M. persicus* from Tehran (Telo) and a group of 10 *Meriones* from Kurdistan (Akanlu) were selected as the control group that did not receive the bacteria. Each of the *M. persicus* was placed in a separate glass cylinder. After 15 d, sterile swab samples were taken from their stool of the rectum, and their bacteria were examined. Following that, 200 *Xenopsylla* fleas infected with *Yersinia pestis* strains of PKR775 were released near each of the *Meriones* studied, and their mortality from plague was assessed.

### Ecology

The ecological conditions of 100 nests, 50 from Telo and 50 from Akanlu, were also investigated (39). The channel diameter of the nests of *Meriones* and the temperature and humidity inside and outside the nests were measured. To study the plant ecosystem in Telo and Akanlu areas, all parts (root, stems, leaves, and flowers) of the plants existing in the regions were collected and transferred to the laboratory for the identification of the species.

### Statistical analysis

The data were analyzed using SPSS software ver. 16 (SPSS, Inc., Chicago, IL). The descriptive (mean and frequency) and analytical analyses (Chi-squared and two independent sample proportion test) were done on the data. P-value less than 0.05 were considered as statistically significant.

## Results

The total number of *M. persicus* was 580 (380 from Telo and 200 from Akanlu). There was no significant difference between the *Meriones* of the two regions studied regarding their physiological characteristics, i.e. body temperature, heart rate, respiratory rate, and feed rate during 24 h, and morphological characteristics, i.e. head and body length, the size of ear, tail, and rear legs, and the color of hair and incisors.

No differences were observed in the blood characteristics of the groups: serum, and alpha-1-antitrypsin pattern.

The cytogenetic and chromosomal analyses indicated that none of the studied *M. persicus* had chromosomal abnormalities. The number of chromosomes in all of the samples studied was forty-two. Non-sex chromosomes were 24 metacentric, 10 sub-metacentric, and 6 acrocentric manners. Sex chromosomes were X and Y chromosomes. Female *Meriones* had two metacentric X chromosomes, while the male *Meriones* had X and Y chromosomes.

According to the results of passive micro-hemagglutination, none of the studied *M. persicus* showed any plague antibodies.

The serum albumin and alpha-2 globulin levels in the *Meriones* of Telo and Akanlu were not significantly different. There was not a significant difference between the levels of IgA and IgG in the serum of *M. persicus* in Akanlu and Telo regions. However, a significant difference was observed between the two groups in terms of the immune diffusion rate of IgA and IgG (*P*<0.0001). Moreover, there was a significant difference between the two regions in terms of the immune diffusion rate ceruloplasmin (*P*<0.0001).

The results of complement variety showed three phenotypes, namely S-S, F-F, and S-F, in the *Meriones* in both regions. There was no difference regarding F-F and S-S phenotypes in Akanlu and Telo; however, S-F phenotype was different in both regions.

*M. persicus* in both areas did not differ in terms of intestinal parasite species, but the percentage of contamination was statistically different (*P*<0.0001), Contamination percentage was higher in *Meriones* of Akanlu. *Moniliformis moniliformis*, *Hymenolepis nana*, and *Trichuris leparis* were observed as intestinal parasites in both regions (Table 1). Nine species of fleas were collected from the bodies of *M. persicus* in both areas. Four species were common in both groups, including *Amphipsylla schelkovnikovi*, *Echidnophaga oschanini*, *Nosopsyllus iranus*, and *Xenopsylla buxtoni*. Four species were observed only on the skins of the *Meriones* in Akanlu, including *Ctenophthalmus dolichus kurdensis*, *Ophthalmopsylla volgensis*, *Paradox-opsyllus grenieri*, and *Rhadinopsylla ucrainica*. *Xenopsylla nuttalli* was only observed on the skins of the *Meriones* in Telo. Haptoglobin was monomorphe in both groups of *M. persicus*. There was not any difference between the *M. persicus* of both areas; the *M. persicus* of both areas were monomorphic and of the C-C type.

**Table 1:** Comparison of serum proteins, immunoglobulins, ceruloplasmin, complement variety, and intestinal parasites between the Meriones of the two regions

***Tested variable***	***Average (range) or Number (%)***		***P-value***
	**Akanlu**	**Telo**	
	n=34	n=48	
Albumin	46(36–65)	45(22–56)	-
Alpha-2 globulin	25(12–37)	23(12–52)	-
Beta and gamma globulin^*^	43(7–39)	31(20–44)	-
Igg	180.4(37–306)	186.6(18–306)	-
Iga	37.3(9–69.2)	41.1(12–69.2)	-
CP	20.88(8–26)	19.39(5.2–26)	-
Migration rate	N=34	N=48	
Albumin	40%	45%	0.65
Alpha-2 globulin	22%	24%	0.83
Beta and gamma globulin^*^	38%	31%	0.50
IgG^*^	100 (75%)	100 (48%)	P<0.001
IgA^*^	100(75%)	100 (49%)	P<0.001
CP^*^	100 (75%)	100 (46%)	P<0.001
Phenotype(n=77)	N=51	N=26	
S-S	41(80%)	23(88%)	0.38
S-F	8(16%)	2(8%)	0.32
F-F	2(4%)	1 (4%)	∼1
Intestinal parasite	N=72	N=129	
Contamination^*^	40(55.5%)	15(11.6%)	P<0.001

* Beta and gamma globulin showed a single band and were not separable from each other.

The electrophoresis pattern of the enzymes in red blood cells, including AP, ESD, 6-PGD, PGM1, and AK was not different among the *Meriones* of both regions.

In Kurdistan, the plant diversity was higher, and the majority of these plants had not been observed in Telo. Comparison of the ecological status of *M. persicus* in Telo and Akanlu demonstrated that heat and moisture were different in the two regions. In addition, the average nest depth of *M. persicus* in Akanlu and Telo was 4.5 and 1.5 meters, respectively. The results of *Meriones* sensitivity to *Yersinia pestis* revealed that 10^−1^ dilution of 10^8^ CFU/ml in the *Meriones* in the Telo region was associated with 100% mortality, but this dilution in the *Meriones* in the Akanlu region resulted in 20% mortality. Moreover, the 10^−4^, 10^−5^, and 10^−6^ dilutions of microbial solution were equal to 70%, 60%, and 20% mortality in the *Meriones* in the Telo region, respectively. These dilutions did not make any mortality in the *Meriones* in the Akanlu region (Table 2).

**Table 2:** The sensitivity of *Meriones* toward microbial solution of *Yersinia pestis* in the two areas studied

***Bacterial Concentration***	***Telo* Dead(%), n=10**	***Akanlu* Dead(%), n=10**	***P-value***
10^−1^	100	20	<0.001
10^−2^	90	10	<0.001
10^−3^	60	10	<0.001
10^−4^	70	0	<0.001
10^−5^	60	0	<0.001
10^−6^	20	0	< 0.001

The isolation of intestinal microbial flora detected 13 bacteria in the intestines of the *Meriones* in Akanlu. These bacteria did not exist in the intestines of the *Meriones* in Telo. A suspension equal to 3×10^8^ CFU/ml was prepared from each of the aforementioned intestinal bacteria and was fed to a group of 12 *Meriones* in Telo. After 15 d, the bacteria that were fed to these *Meriones* were colonized in their intestine and identified by standard biochemical techniques.

The *Meriones* and their mortality were assessed by means of 200 *Xenopsylla* fleas infected with *Yersinia pestis* strain of PKR775. The dead *Meriones* were autopsied. In addition, the plague bacillus was isolated from their spleen and confirmed by bacteriophage cultures. The resistant *Meriones* were killed and their spleens were examined for the plague infection. In none of them, the plague infection was isolated. Among the 13 types of bacteria isolated from the intestines of the *Meriones* in Kurdistan, seven types induced more than 80% resistance to plague bacillus in the *Meriones* of Tehran (Table 3).

**Table 3:** The effects of inducing cross-resistance to plague in bacteria isolated from the intestines of the *Meriones* in Akanlu fed to the *Meriones* in Telo

***Bacterium***	***No. of Meriones***	***Mortality***	***Resistance***
*Streptococcus faecalis*	12	8	33.4
*Klebsiella Spp*	12	8	33.4
*Streptococcus mitis*	12	6	50
*Staphylococcus albus*	12	6	50
*Hafinia Spp*	12	5	58.4
*Citrobacter Spp*	12	4	66.7
*Streptococcus sanginus*	12	2	83.4
*Serratia marcscens*	12	2	83.4
*Bacillus pasterii*	12	2	83.4
*Bacillus mycoides*	12	2	83.4
*Enterobacter agglomerans*	12	0	100
*Corynebactrium xerosis*	12	0	100
*Bacillus alvei*	12	0	100
Control			
Telo	12	12	0
Akanlu	10	0	100

## Discussion

There was no significant difference between the *Meriones* of the two studied regions in terms of physiological characteristics, i.e. body temperature, heart rate, respiratory rate, and feed rate during 24 h, and morphological characteristics such as head and body length, the size of ear, tail, and rear legs, and the color of hair and incisors. No differences were observed in the characteristics of the blood groups, serum, and alpha-1-antitrypsin pattern.

The present study was conducted about 40 years ago, and at that time, the effect of cytokines on the immune system was not well comprehended. *Meriones* microflora in the Akanlu region may also lead to inducing cytokines such as IL-22 and IL-17, which could cause the resistance to plague by provoking the host immune responses. The same kind of resistance is created by transferring the *Meriones* microflora in Akanlu to the *Meriones* in Telo. Another important immune response is neutrophil cytotoxicity that is increased by peptidoglycan (gram-positive bacteria) microbiota in systemic infections (40). Eight out of 13 bacteria isolated from the intestinal microflora of the *Meriones* in Akanlu were gram-positive bacteria.

For the first time, the serum proteins in both regions were studied. The levels of IgA and IgG were significantly higher in the *Meriones* of Akanlu in comparison with those of the *Meriones* in Telo. By considering the vital role of each of these factors, a higher level of *M. persicus* in Akanlu could enhance the resistance to plague (22, 41).

The blood ceruloplasmin level in the *Meriones* of Akanlu is significantly higher than that of the *Meriones* in Telo. By considering the effect of ceruloplasmin-transmission of copper to tissues, its important role in the metabolism of iron, and the crucial role of iron in the pathogenicity of *Yersinia pestis*, the higher resistance to plague observed amongst the *Meriones* of Akanlu to plague could be due to the high levels of ceruloplasmin in their blood (42). The importance and the biologic role of complements in cell lysis and microbial destruction were also noted. The phenotype of S-F in Akanlu could be another reason for the high resistance of *Meriones* to plague.

In addition, four types of fleas were isolated from *M. persicus* in Akanlu, while they were not found in the Telo region. This is a significant observation due to the relationship between the vector arthropods and their reservoirs. With regard to various lifespans and other biologic characteristics amongst different types of flea, these fleas could play a crucial role in the maintenance of plague in Akanlu.

Based on the ecosystem and the list of the known plants (43), there was a significant difference between Akanlu and Telo regions. Since there were differences between the *Meriones* of these two regions regarding blood and serum factors, a number of plants in Akanlu, which are a part of the diet of these animals, could be attributed to the differences in increasing or decreasing the blood factors. Future studies investigate the role of these plants.

Furthermore, the nest of *M. persicus* in Akanlu was deeper in comparison with that of *M. persicus* in Telo. This difference is of utmost importance since changes in temperature in winter in Akanlu would not affect plague microbes very much in terms of coldness or dryness; therefore, these microorganisms can live longer. This finding was also reported in another study that reported the soil in these nests could keep the plague bacteria alive for more than a year (44). As this study was conducted about 40 years ago, some of the materials and methods used in this investigation are old-fashioned; therefore, conducting similar studies with updated tools could result in more findings that are precise.

The bacteria of the intestinal micro flora of *M. persicus* in Akanlu could play a role in relative resistance to plague amongst the *Meriones* in Kurdistan, the transmission of certain bacteria in the intestines of *M. persicu*s in Akanlu can lead to the resistance against plague amongst the *Meriones* of the Telo region. This transmission could significantly decrease the rate of death amongst *M.* after exposure to *Yersinia pestis*-infected fleas.

The beneficial effects of intestinal microflora on mammals and human health have been the subject of several studies (45–47). Intestinal microflora contributes to supplying the essential nutrition and improving the metabolism of non-digestible substances in the host body. However, one of the most important roles of intestinal microflora is modulating the host immune system to fight against infectious diseases (48). The microbiota composition plays a key role in the development of resistance or susceptibility to infections. In addition, oral transmission of microbiota from susceptible mice to a kind of infection can cause the same kind of sensibility in animals that were not previously susceptible to infection. Moreover, microbiota transmission from a resistant animal to a sensitive one can create the same type of resistance in the sensitive animal, thus reducing the colonization of the pathogens and the mortality rate. This resistance to infectious diseases is associated with the overexpression of IL-22. This cytokine can lead to the proliferation of IL-17 by producing T helper cells as well as anti-microbial peptides, including Reg3γ and Reg3β, which enhances the activities of the host immune system against pathogens (49). In animal models, the absence of these cytokines (IL-22) may lead to sensitivity to infection (50).

## Conclusion

Various factors could affect the sensitivity to plague among the *M.* Various blood factors could influence the sensitivity toward plague, and resistance to plague could be transmitted from the resistant *M.* to the sensitive ones by fecal or blood transplants.

## Ethical considerations

Ethical issues (Including plagiarism, informed consent, misconduct, data fabrication and/or falsification, double publication and/or submission, redundancy, etc.) have been completely observed by the authors.

## References

[1] Finney CA Kamhawi S Wasmuth JD (2015). Does the arthropod microbiota impact the establishment of vector-borne diseases in mammalian hosts? PLoS Pathog, 11:e1004646. 25856431 10.1371/journal.ppat.1004646 PMC4391854

[2] Weldon L Abolins S Lenzi L (2015). The gut microbiota of wild mice. PLoS One, 10:e0134643. 26258484 10.1371/journal.pone.0134643 PMC4530874

[3] Mosca A Leclerc M Hugot JP (2016). Gut microbiota diversity and human diseases: should we reintroduce key predators in our ecosystem? Front Microbiol, 7: 455. 27065999 10.3389/fmicb.2016.00455 PMC4815357

[4] McKenney PT Pamer EG (2015). From hype to hope: the gut microbiota in enteric infectious disease. Cell, 163: 1326–1332. 26638069 10.1016/j.cell.2015.11.032 PMC4672394

[5] Van Den Elsen LW Poyntz HC Weyrich LS (2017). Embracing the gut microbiota: the new frontier for inflammatory and infectious diseases. Clin Transl Immunology, 6: e125. 28197336 10.1038/cti.2016.91 PMC5292562

[6] Esamaeili S Azadmanesh K Naddaf SR (2013). Serologic survey of plague in animals, Western Iran. Emerg Infect Dis, 19 (9): 1549–1551. 10.3201/eid1909.121829 PMC3810907 23968721

[7] Hashemi Shahraki A Carniel E Mostafavi E (2016). Plague in Iran: its history and current status. Epidemiol Health, 38: e2016033 27457063 10.4178/epih.e2016033 PMC5037359

[8] Baltazard M Seydian B Mofidi C Bahmanyar M Pournaki R (1953). [The resistance of certain wild rodents to plague. I. Facts observed in nature]. Ann Inst Pasteur (Paris). 85: 411–442. 13148779

[9] Ellerman J (1941). The families and genera of living rodents. Volume II Muridae. British Museum Inc, England, pp.: 1–690

[10] Shar S Lkhagvasuren D Molur S (2016). Rhombomys opimus (errata version published in 2017). The IUCN Red List of Threatened Species, 2016: e.T19686A115153015.

[11] Tjio J Whang J (1962). Chromosome Preparatons of Bone Marrow Cells without Prior In Vitro Culture or In Vivo Colchicine Administration. Stain Technol, 37: 17–20. 13921436 10.3109/10520296209114563

[12] Yunis JJ Sanchez O (1975). The G-banded prophase chromosomes of man. Humangenetik, 27 (3): 167–172. 50274 10.1007/BF00278343

[13] Buckton KE Evans HJ (1973). Methods for the analysis of human chromosome aberrations. World Health Organization Inc, Geneva pp. 60.

[14] Keyvanfar A (1962). Sérologie et immunologie de deux espèces de thonidés (Germo alalunga Gmelin et Thunnus thynnus Linné) de l’Atlantique et de la Méditerranée. Revue des Travaux de l’Institut des Pêches Maritimes, 26 (4): 407–456.

[15] Cavanaugh D Deoras P Hunter D Marshall J Jr (1970). Some observations on the necessity for serological testing of rodent sera for Pasteurella pestis antibody in a plague control programme. Bull World Health Organ, 42 (3): 451–459. 5310211 PMC2427534

[16] Suzuki S Chikasato Y Hotta S (1974). Studies on antiplague haemagglutinating antibodies: Treatment of test sera with acetone and 2-mercaptoethanol. Bull World Health Organ, 51 (3): 237–243. 4549347 PMC2366276

[17] Davis D Heisch R McNeill D Meyer K (1968). Serological survey of plague in rodents and other small mammals in Kenya. Trans R Soc Trop Med Hyg, 62 (6): 838–861. 5729573 10.1016/0035-9203(68)90013-8

[18] Leonardy J (1971). Serum protein electrophoresis in office practice. South Med J, 64 (2): 129–137. 4100143 10.1097/00007611-197102000-00001

[19] Bock E (1978). Immunoglobulins, prealbumin, transferrin, albumin, and alpha2-macroglobulin in cerebrospinal fluid and serum in schizophrenic patients. Birth Defects Orig Artic Ser, 14 (5):283–295. 80238

[20] Schwick HG Störiko K Becker W (1969). Qualitative determination of plasma proteins by immunoprecipitation. Behring Diagnostics Inc, USA, pp. 1–40.

[21] Daneshmand P Farhud D (1990). Alpha-1-antitrypsin types and serum levels in toxoplasmosis. Hum Hered, 40 (2): 116–117. 2335366 10.1159/000153916

[22] Smithies O (1955). Zone electrophoresis in starch gels: group variations in the serum proteins of normal human adults. Biochem J, 61 (4): 629–641. 13276348 10.1042/bj0610629 PMC1215845

[23] Farhoud D (1979). Use of transferrin polymorphism in legal medicine and paternity disputed cases Iran J Public Health, 8 (1): 1–2.

[24] Farhud D Sadighi H Amirshahi P Tavakkoli F (1993). Serum level measurements of Gc, Cp, IgG, IgA and IgM in patients with Favism in Iran. Iran J Public Health, 22 (1): 1–4.

[25] Porter RR (1958). Separation and isolation of fractions of rabbit gamma-globulin containing the antibody and antigenic combining sites. Nature, 182 (4636): 670–1. 13590068 10.1038/182670a0

[26] Steinbuch M Audran R (1969). The isolation of IgG from mammalian sera with the aid of caprylic acid. Arch Biochem Biophys, 134 (2): 279–284. 4982185 10.1016/0003-9861(69)90285-9

[27] Hopkinson D Spencer N Harris H (1963). Red cell acid phosphatase variants: a new human polymorphism. Nature, 199:969–971. 14073798 10.1038/199969a0

[28] Parkin B Adams EG (1975). The typing of esterase D in human bloodstains. Med Sci Law, 15 (2): 102–105. 1160553 10.1177/002580247501500204

[29] Fitch L Parr C (1966). Development of zymograms by paint brush technique. Biochem J, 99 (1): 1–20.

[30] Hopkinson D Spencer N Harris H (1964). Genetical studies on human red cell acid phosphatase. Am J Hum Genet, 16: 141–154. 14131868 PMC1932462

[31] Bowman JE Frischer H Ajmar F Carson PE Gower MK (1967). Population, family and biochemical investigation of human adenylate kinase polymorphism. Nature, 214 (5093): 1156–8. 6053088 10.1038/2141156a0

[32] Spencer N Hopkinson D Harris H (1964). Phosphoglucomutase polymorphism in man. Nature, 204: 742–5. 14235665 10.1038/204742a0

[33] Gordon R. M (1956). Insects of medical importance. Br Med J, 2 (5001): 1103.

[34] Farhang-Azid A (1969). The flea fauna of Iran. V. Fleas collected from Khorasan Ostan from 1965 to 1967. Bull Soc Pathol Exot Filiales, 62 (1): 153–8. 5395501

[35] Brumpt É (1949). Precis Parasitology. 6th ed Masson Inc, France, pp: 1–1042.

[36] Yamaguti S (1959). Systema Helminthum. vol. II The cestodes of vertebrates. Interscience Inc, USA, pp: 1–860

[37] Reed LJ Muench H (1938). A simple method of estimating fifty per cent endpoints. Am J Epidemiol, 27 (3): 493–497.

[38] Reading CA Glynn LE (1981). Gradwohl’s Clinical Laboratory Methods and Diagnosis. Immunology, 43 (4): 803.

[39] Misonne X (1959). The rodents of the areas of the Congolese plaguel. Ann Soc Belg Med Trop (1920), 39: 437–493. 13848661

[40] Clarke TB Davis KM Lysenko ES (2010). Recognition of peptidoglycan from the microbiota by Nod1 enhances systemic innate immunity. Nat Med, 16 (2): 228–231. 20081863 10.1038/nm.2087 PMC4497535

[41] Lubet MT Kettman JR (1978). Primary antibody and delayed type hypersensitivity response of mice to ovalbumin. Immunogenetics, 6 (1): 69–79.

[42] Carniel E (2001). The Yersinia high-pathogenicity island: an iron-uptake island. Microbes Infect, 3 (7): 561–9. 11418330 10.1016/s1286-4579(01)01412-5

[43] Golvan Y Rioux J (1961). Ecologie des mérions du Kurdistan Iranien: relations avec l’épidémiologie de la peste rurale. Ann Parasit Hum Comp, 36 (4): 449–558. 13900030

[44] Karimi Y (1963). [NATURAL PRESERVATION OF PLAGUE IN SOIL]. Bull Soc Pathol Exot Filiales, 56: 1183–1186. 14153921

[45] Drissi F Raoult D Merhej V (2017). Metabolic role of lactobacilli in weight modification in humans and animals. Microb Pathog, 106: 182–194. 27033001 10.1016/j.micpath.2016.03.006

[46] Kinross JM Darzi AW Nicholson JK (2011). Gut microbiome-host interactions in health and disease. Genome Med, 3 (3): 14. 21392406 10.1186/gm228 PMC3092099

[47] Lozupone CA Stombaugh JI Gordon JI (2012). Diversity, stability and resilience of the human gut microbiota. Nature, 489 (7415): 220–230. 22972295 10.1038/nature11550 PMC3577372

[48] Cash HL Whitham CV Behrendt CL Hooper LV (2006). Symbiotic bacteria direct expression of an intestinal bactericidal lectin. Science, 313 (5790): 1126–1130. 16931762 10.1126/science.1127119 PMC2716667

[49] Willing BP Vacharaksa A Croxen M (2011). Altering host resistance to infections through microbial transplantation. PLoS One, 6 (10): e26988. 22046427 10.1371/journal.pone.0026988 PMC3203939

[50] Zheng Y Valdez PA Danilenko DM (2008). Interleukin-22 mediates early host defense against attaching and effacing bacterial pathogens. Nat Med, 14 (3): 282–9. 18264109 10.1038/nm1720

